# Membrane-Active Sequences within gp41 Membrane Proximal External Region (MPER) Modulate MPER-Containing Peptidyl Fusion Inhibitor Activity and the Biosynthesis of HIV-1 Structural Proteins

**DOI:** 10.1371/journal.pone.0134851

**Published:** 2015-07-31

**Authors:** Si Min Zhang, Alenka Jejcic, James P. Tam, Anders Vahlne

**Affiliations:** 1 Division of Clinical Microbiology, Department of Laboratory Medicine, Karolinska Institutet, Stockholm, SE-141 86, Sweden; 2 School of Biological Sciences, Nanyang Technological University, 60 Nanyang Drive, Singapore, 637551, Singapore; 3 Department of Microbiology and Immunology, Rega Institute for Medical Research, KU Leuven, Minderbroederstraat 10, Leuven, B-3000, Belgium; Chinese Academy of Medical Sciences, CHINA

## Abstract

The membrane proximal external region (MPER) is a highly conserved membrane-active region located at the juxtamembrane positions within class I viral fusion glycoproteins and essential for membrane fusion events during viral entry. The MPER in the human immunodeficiency virus type I (HIV-1) envelope protein (Env) interacts with the lipid bilayers through a cluster of tryptophan (Trp) residues and a C-terminal cholesterol-interacting motif. The inclusion of the MPER N-terminal sequence contributes to the membrane reactivity and anti-viral efficacy of the first two anti-HIV peptidyl fusion inhibitors T20 and T1249. As a type I transmembrane protein, Env also interacts with the cellular membranes during its biosynthesis and trafficking. Here we investigated the roles of MPER membrane-active sequences during both viral entry and assembly, specifically, their roles in the design of peptidyl fusion inhibitors and the biosynthesis of viral structural proteins. We found that elimination of the membrane-active elements in MPER peptides, namely, penta Trp→alanine (Ala) substitutions and the disruption of the C-terminal cholesterol-interacting motif through deletion inhibited the anti-viral effect against the pseudotyped HIV-1. Furthermore, as compared to C-terminal dimerization, N-terminal dimerization of MPER peptides and N-terminal extension with five helix-forming residues enhanced their anti-viral efficacy substantially. The secondary structure study revealed that the penta-Trp→Ala substitutions also increased the helical content in the MPER sequence, which prompted us to study the biological relevance of such mutations in pre-fusion Env. We observed that Ala mutations of Trp664, Trp668 and Trp670 in MPER moderately lowered the intracellular and intraviral contents of Env while significantly elevating the content of another viral structural protein, p55/Gag and its derivative p24/capsid. The data suggest a role of the gp41 MPER in the membrane-reactive events during both viral entry and budding, and provide insights into the future development of anti-viral therapeutics.

## Introduction

**Fig 1:**
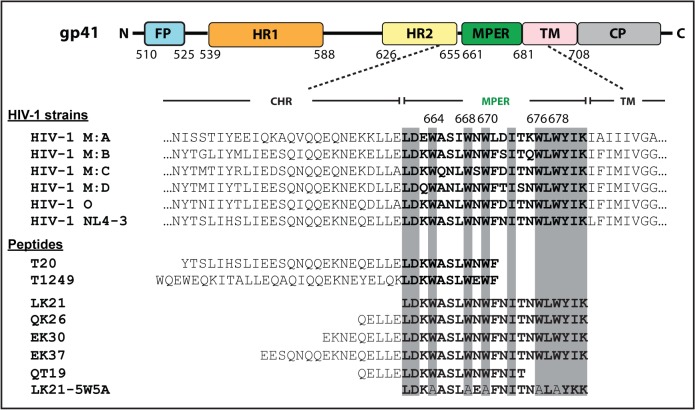
Schematic representation of HIV-1 gp41 and partial sequence alignment of the gp41 from different groups. Partial HR2, the MPER and the TMD sequences of HIV-1 group M subtypes A, B, C and D; group O and the experimental strain of this study, HIV(NL4-3) were aligned[15]. Sequences of the anti-HIV-1 first and second generation fusion inhibitor, T20 and T1498, respectively, are shown together with the MPER-containing peptides tested in this study, EK37, EL30, QK26, QT19, LK21 and LK21-5W5A, and all are aligned with the MPER sequence. The MPER sequence is highlighted in bold with its conserved residues shaded. Peptide LK21-5W5A have all five tryptophan residues in MPER sequence substituted by Ala.

The envelope protein (Env) of human immunodeficiency virus type I (HIV-1) is a class I fusion glycoprotein [1]. It protrudes out of the viral envelope as homotrimers composed of non-covalently-linked gp120/gp41 heterodimers [2–4]. Recognition of the viral receptor and co-receptor by the surface gp120 subunit activates the fusion machinery in the transmembrane (TM) gp41 subunit (Fig 1) [5–8], resulting in the insertion of gp41 N-terminal fusion peptide region (FP) into the target cell membrane. This pre-fusion intermediate conformation of gp41 connects the cellular membrane and the viral envelope, exposing and extending the two heptad repeat (HR) regions, HR1 and HR2 [9–11]. The intermediate conformation quickly resolves into a stable six-helix bundle (6-HB) conformation, after HR2 folds back onto the central HR1 to form a coiled-coil trimer-of-dimers [12, 13]. This predisposes the opposing membranes into sufficient proximity for subsequent envelope fusion with the plasma membrane and viral content delivery [14].

The post-6-HB-formation lipid mixing and subsequent membrane fusion is mediated by the membrane proximal external region (MPER) in gp41 (Fig 1), a hydrophobic region between HR2 and the TM domain [16, 17]. MPER induces fusion-required membrane perturbation through a direct interaction with the membranes [18, 19]. Sequential alignments have revealed a high conservation of MPER among different groups of HIV-1 (Fig 1) [15]. In particular, it contains two conserved sequence elements that contribute to its membrane perturbation function. One is an enrichment of aromatic amino acids, in particular Trp (Fig 1), and the other is its cholesterol-interacting C-terminus.

Previous studies have shown that Ala substitutions of the five Trp residues abrogated the ability of MPER-containing peptides to partition into and destabilize liposomal membranes [18, 19]. In the context of gp41, the Trp→Ala substitutions in MPER inhibit membrane fusion events, such as the fusion pore expansion, during viral entry. [17]. Furthermore, the MPER C-terminus (LWYIK) shows sequence characteristics of the cholesterol recognition/interaction amino acid consensus (CRAC) motif,-L/V-(X)(1–5)-Y-(X)(1–5)-R/K- [20, 21]. Cholesterol is enriched in viral envelopes and also in the cell membrane lipid rafts where viral receptors are enriched during viral entry, making the membranes rigid and counteracting viral entry [22, 23]. The C terminus of MPER can facilitate the induction of membrane destabilization and subsequent fusion in the cholesterol-enriched liposomal membranes [24–26].

The membrane destabilizing ability of MPER sequences has implication in designing viral fusion inhibitors. T20 and T1249, two of the first fusion inhibitors, are synthetic peptides containing sequences from both the HR2 and the N-terminus of MPER (Fig 1) [27, 28]. HR2 sequences enable these inhibitors to bind to the exposed HR1 of gp41 in the pre-fusion intermediate conformation, and thereby halt the formation of the 6-HB [9]. In addition, MPER sequences act as inhibitors at a later stage of the viral entry, possibly through anchoring the peptide into the cellular membrane through their last four residues, WNWF [29–31]. The membrane anchoring abilities of the two fusion inhibitors correlate with their antiviral activity [32]. Still, peptide fusion inhibitors, such as T20 and T1249, only included partial MPER sequences without the C-terminal sequence [33]. Due to the high variation and mutation rate of HIV-1 Env protein, the fusion inhibitors were constantly challenged by drug resistance issues. The MPER is a conserved, exposed and accessible region, and therefore it could be an additional target for the design of potential fusion inhibitors.

Apart from its role in viral entry, Env also interacts with host cellular membranes during its biosynthesis and trafficking to the viral budding site. As a type I transmembrane protein, Env is co-translationally translocated into the rough endoplasmic reticulum (ER) and further transported to the Golgi complex for maturation into gp160 and subsequent proteolytic processing [34, 35]. The resulting gp120/gp41 trimers are then transported to the cholesterol-rich plasma membrane regions (e.g. lipid rafts), following the secretory pathway [36]. Studying SARS-CoV (severe respiratory syndrome associated corona virus) we observed that the Trp residues in MPER modulate the selective incorporation of the spike protein into lipid rafts (unpublished observations, See S1 and S2 Figs). However, Salzwedel and colleagues found that neither deletion nor Trp substitution mutations in the HIV-1 MPER affected Env maturation, or steady-state levels, but had an effect on its incorporation into virus particles [17].

Here we describe the roles of the Trp residues in the membrane-active MPER sequence in anti-HIV fusion inhibitor design and a surprising role in the biosynthesis of viral structural proteins. Six peptides ranging from 19 to 37 amino acids (a.a.) were designed to contain the MPER sequence in its full length, with C-terminal truncation, or with penta Trp→Ala substitution (Fig 1). Their anti-viral activities were tested in a single-round infectivity assay using pseudovirus. Dimerization of anti-viral peptides has been shown to enhance both their structural stability and the number of interaction sites and thus their anti-viral efficacy [37–40]. Therefore, we also tested our peptides as N- or C-terminal dimers. HIV-1 Env with mutations of Trp residues at the MPER region were also constructed to examine the roles of the Trp residues in the biosynthesis, maturation, trafficking, and viral incorporation of the viral structural proteins.

## Materials and Methods

### Reagents and antibodies

HIV-1 gp41-derived monomeric peptides were custom synthesized by Synpeptide Co Ltd (Shanghai, China) and the dimeric peptides by Pepscan Presto BV (Amsterdam, Netherlands). The antibody to gp160/41 (Chessie 8) and Vif (#319) were obtained through the NIH AIDS Research and Reference Reagent Program [41]. Polyclonal rabbit anti-Nef antibody was obtained from Thermo Fisher Scientific, Inc. Monoclonal mouse anti-β-actin antibody was obtained from Sigma. The antibody to p24 (EF7), has previously been described [42]. The secondary antibodies, HRP-conjugated polyclonal goat anti-rabbit immunoglobulins and HRP-conjugated polyclonal rabbit anti-mouse immunoglobulins, were obtained from DAKO.

### Cell lines and plasmids

HLtat (Cat. #1293) and TZM-bl (Cat. #8129) cell lines were obtained through NIH AIDS Research and Reference Reagent Program [43–47] and cultured in Dulbecco's Modified Eagle Medium (Life technologies) supplemented with 10% fetal bovine serum (Life technologies) and 1% Penicillin-Streptomycin (Life technologies). The expression plasmid for Env, Nef (negative regulator factor) and Vpu (virion protein U) from the HIV-1 strain NL4-3 (pNL1.5EU+) was kindly provided by Dr. Stefan Schwartz (Lund University, Lund, Sweden) [48].

pNLHIVxΔuΔss contains a *vpu*-deficient HIV-1 genome with the signal sequence of *env* truncated and was constructed from pNL4-3 with Quick Change II XL (Stratagene) [49]. Specifically, the fragment EcoRI-BamHI from pNL4-3 was cloned into pUC18 to create pUC18EnvxΔu that has the Vpu start codon mutated to ATA and contains a Xma I cleavage site immediately upstream of Vpu, using the primer pair B(xΔu): (forward) 5’-GCAGTAAGTAGTAC**CC**G**GG**AT**A**CAACCTATAATAGTAGCAATAG-3’, (reverse) 5’-CTATTGCTACTATTATAGGTTGTATCCCGGTACTACTTACTGC-3’. The same mutation was introduced into ΔnSS-gp160 [50], encoding Vpu and a gp160 with a signal peptide of 11 a.a. length, using the primer pair A(xΔu): (forward) 5’-GAAGCGCGCACGGAGTAC**CC**G**GG**AT**A**CAACCTATAATAGTAGC-3’, (reverse) 5’-GCTACTATTATAGGTTGTATCCCGGGTACTCCGTGCGCGCTTC-3’. The XmaI-NheI fragment of the constructed mutant ΔnSS-gp160 was then directionally inserted into pUCEnvxΔu to create pUCEnvxΔuss11, in which site-directed mutagenesis was carried out with primer pair(Δss): (forward) 5’-GGATATTGAT**A**ATCTGTAGTGCTA**TG**GAAAAATTGTGGGTC-3’, (reverse) 5’-GACCCACAATTTTTCCATAGCACTACAGATTATCAATATCC-3’ to introduce gp160 signal sequence mutation. The EcoRI-NheI fragment of the resulted mutant construct was then subcloned into p83-10 [51], of which the SaII-NcoI fragment was directionally transferred into pNL4-3, creating pNLHIVxΔuΔss.

pNL1.5EU+W5A, pNL1.5EU+W3A and pNL1.5+W2A were constructed from pNL1.5EU+, using Quick Change II (Agilent Technologies). pNL1.5EU+W2A was constructed using the primer pair A(W676.678): (forward) 5’-GCAAGTTTGTGGAATTGGTTTAACATAACAAAT**GC**GCTG**GC**GTATATAAAATTATTCATAATGATAGTAGGAGG-3’, (reverse) 5’- CCTCCTACTATCATTATGAATAATTTTATATAC**GC**CAGC**GC**ATTTGTTATGTTAAACCAATTCCACAAACTTGC -3’. pNL1.5EU+W3A was constructed using the primer pair B(W664.668.670): (forward) 5’- GAATGAACAAGAATTATTGGAATTAGATAAA**GC**GGCAAGTTTG**GC**GAAT**GC**GTTTAACATAACAAATTGGCTGTGGTA -3’, (reverse) 5’- TACCACACCACATTTGTTATGTTAAAC**GC**ATTC**GC**CAAACTTGCC**GC**TTTATCTAATTCCAATAATTCTTGTTCATTC -3’. pNL1.5EU+W5A was constructed from pNL1.5EU+W3A using primer pair A (W676.678). The mutated pNL1.5EU+ plasmids were sequenced between nucleotide 7988 to 8533 with the sequencing primer (7988F-CTCCTGGGGATTTGGGGTTG and 8533R-GTCTCTCAAGCGGTGGTAGC).

### Pseudotyped HIV-1 production and precipitation of virus particles

HLtat cells (5 X 10^5^) were co-transfected with the HIV-1 proviral clone pNLHIVxΔuΔss, and with the Env- and Vpu-expressing plasmid pNL1.5EU+, using FugeneHD (Roche). For the production of mutant pseudotyped HIV-1, we used pNL1.5EU+W5A, pNL1.5EU+W3A or pNL1.5EU+W2A during transfection, instead of pNL1.5EU+. The cell culture supernatant was collected 48 hours (h) post-transfection, clarified by centrifugation and 0.45-μm filtration, used for virion precipitation or stored at -80°C until further use.

Viral particles were precipitated from the clarified and 0.45μm-filtered cell culture supernatant at 4°C for 48h in 1:6 (vol:vol) polyethylene glycol 6000 containing 0.667M NaCl. The precipitated particles were further concentrated by centrifugation at 16,000 times g for 20 min at 4°C. The virus pellets were dissolved in radio-immunoprecipitation assay (RIPA) buffer containing 50 mM Tris-HCL (pH = 7.4), 1% Triton X-100, 1% deoxycholate, 150 mM NaCl, 1 mM EDTA, and 0.1% sodium dodecyl sulfate (SDS) and supplemented with Complete protease inhibitor cocktail (Roche).

### Single-round infectivity assay

Cell-free pseudotyped HIV-1 virus of 100TCID50 was either pre-incubated with peptides of various concentrations at 37°C for 1 h, or applied directly to infect 10^4^ CD4+, CCR5+ TZM-bl cells. TZM-bl cells contain integrated copies of an LTR (long terminal repeats)-driven luciferase reporter gene. Seventy-two h post-infection, the infectivity of pseudovirus was assessed as luciferase activity, using the One-Glo Luciferase assay system (Promega). The TCID50 of pseudotyped HIV-1 was calculated based on the luciferase activity of the infected TZM-bl cells, using the Reed–Muench method and the cut-off value set at 3 times of the background signal [52].

Effective concentrations of peptides inhibiting 50% (IC_50_) and 80% (IC_80_) of viral infectivity were estimated with GraphPad Prism. The IC50 and IC80 values were estimated from the dose-response curves that were curve-fitted with the sigmoidal dose-response non-linear regression model on Prism GraphPad software, using the percentage of inhibition data and the log values of peptide concentrations.

### PrestoBlue cell viability assay on TZM-bl cells

The cytotoxicity effect of the peptides on TZM-bl cells were determined by PrestoBlue cell viability assay (Lifetechnologies, Singapore), according to the manufacturer’s protocol. Briefly, peptides of different concentrations were added to 10,000 TZM-bl cells seeded in 96-well plates. Upon 24 h of incubation, Prestoblue cell viability reagent was added to the cells and incubated for 30 min at 37°C. Resulting absorbance values were recorded at 570nm and 600nm (baseline). Final spectrum was obtained by normalizing the 570 nm values to the 600 nm values.

### Circular dichroism spectroscopy

Circular dichroism spectroscopy analysis was performed to study the secondary structure of the monomeric peptides in trifluoroethanol (TFE). TFE was used to mimic the hydrophobic environment at the membrane fusion junction. The measurements were made on Chirascan circular dichroism spectrometer (Applied Photophysics). Fifty μM peptide was dissolved in 10%, 20%, or 40% TFE and subjected to the measurement with three repeats in a cell of 0.1mm pathlength (Hellma Uk Ltd.) at 25°C. Samples were measured between 190nm and 260nm, with a 0.5nm step resolution, a measurement speed of 60nm/min and a 1nm bandwidth. The baseline was measured with 10% TFE, with three repeats. The final spectrum was generated by subtracting the averaged sample spectrum with the baseline spectrum, followed by smoothing with a Savitsky-Golay filter. The secondary structures of the peptides were estimated from deconvoluting the respective circular dichroism spectra using the CDSSTR deconvolution algorithm on Dichroweb, with a cut-off NRMSD value set at 0.15 [53–55].

### Western blot

HLtat cells (5 X 10^5^) expressing WT or mutant pseudotyped HIV-1 were lysed in RIPA buffer on ice, centrifuged and mixed with Laemmli reducing buffer. Precipitated pseudotyped HIV-1 viral particles dissolved in RIPA buffer were also prepared in Laemmli reducing buffer. Cell lysates or virus lysates were resolved by SDS-polyacrylamide gel electrophoresis (PAGE), transferred to nitro-cellulous membranes and immunoblotted with anti-gp41 antisera and followed by HRP-conjugated anti-rabbit secondary antibody for Env expression, or with anti-p24 antisera and followed by HRP-conjugated anti-rabbit secondary antibody for p24 and p55/Gag expression, or with anti-Nef antibody and followed by HRP-conjugated anti-rabbit secondary antibody for Nef expression, or with anti-Vif and followed by HRP-conjugated anti-mouse secondary antibody for Vif expression, or with anti-β-actin and followed by HRP-conjugated anti-mouse secondary antibody for β-actin expression. The membranes were either exposed to film or analyzed with G:BOX Chemi XX6 (Syngene). The band intensities were quantified by ImageJ software.

### Measurement of intracellular and intraviral p24 levels

p24 levels in the cell-free lysate of the virus-producing HLtat cells or the cell culture supernatants were quantified by the automated system ARCHITECT (Abbott). A standard curve was generated using p24 of known concentration and curve-fitted with the linear non-regression model on Prism Graphpad software. Prior to each measurement, the samples were diluted to the concentrations within the linear range of the standard curve.

## Results

### 1. Sequence requirements of the MPER-containing peptides in inhibiting pseudo-HIV-1 (NL4-3) entry

**Fig 2:**
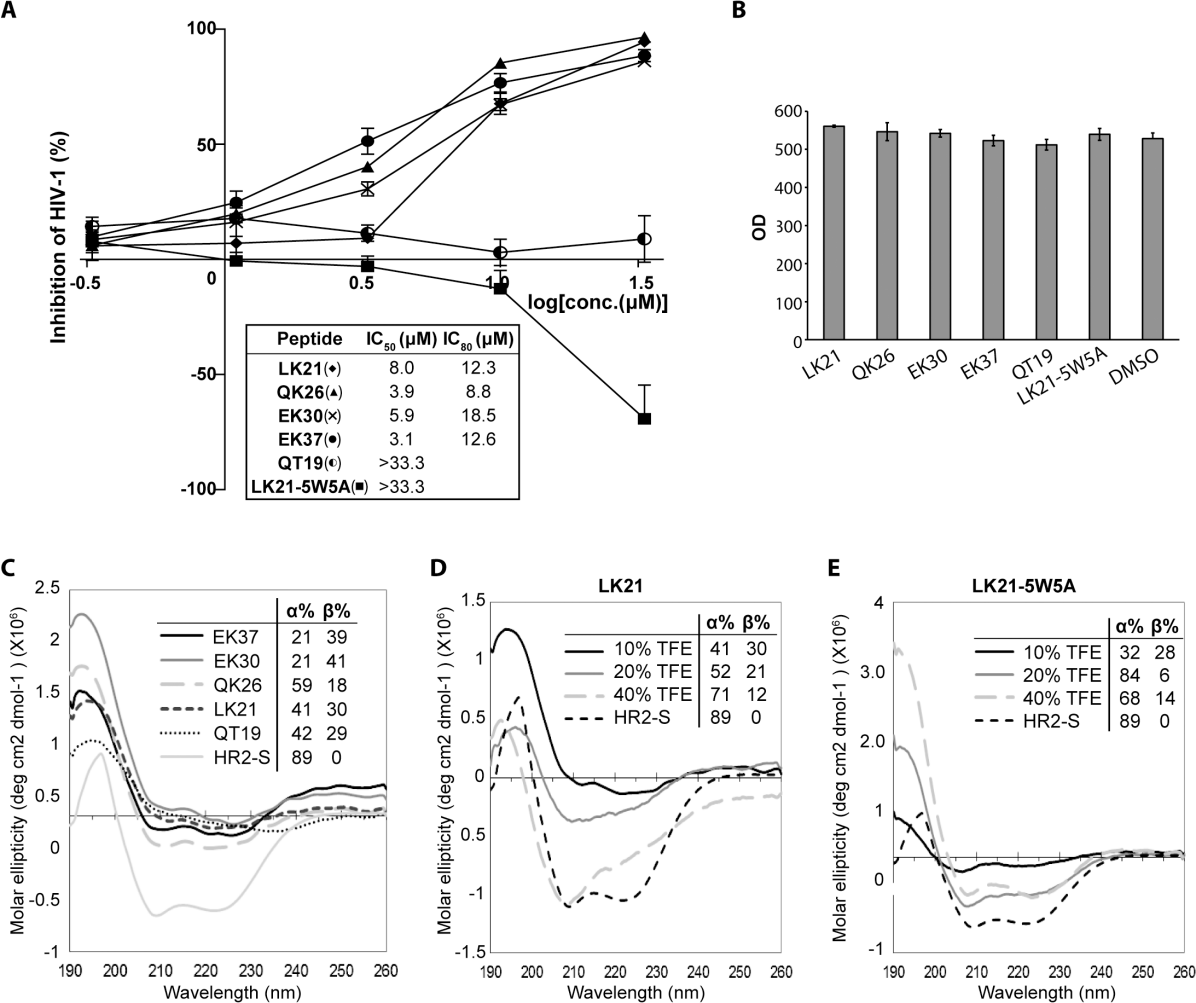
Determination of the sequence requirement and the secondary structures of the MPER-containing peptidyl fusion inhibitors. **A.** Test for inhibition of pseudo HIV-1 (NL4-3) infection of TZM-bl cells by the MPER-containing peptides and the mutant analog LK21-5W5A. Pseudo-HIV-1 virus with concentration of 100TCID50 was incubated with peptides at various concentrations for 1 h, and then added to TZM-bl cells. Seventy-two h post-infection, tat-activated luciferase activity in TZM-bl cells was assessed as a measurement of viral entry and infection. Concentrations of the peptides yielding a 50% and 80% reduction in luciferase activity were estimated with GraphPad Prism. Results shown were summarized from three independent experiments with serial dilutions of peptides in replicates of two. **B.** No cytotoxicity effect was observed for the MPER-derived peptides at 50 μM in TZM-bl cells. Fifty μM of the peptides were incubated with 10,000 Vero cells for 24 h. PrestoBlue cell viability reagent was subsequently added to the cells, and cytotoxicity effects were monitored as absorbance values (OD) at 570 nm and 600 nm (baseline). **C.** Circular dichroism spectra and the estimated secondary structure contents of the peptide LK21, QK26, EK30, EK37 and QT19 in 10% TFE. Fifty μM of the peptides were dissolved in H_2_O supplemented with 10% TFE and were subjected to circular dichroism spectroscopy measurement. **D.** Circular dichroism spectra and the estimated secondary structure contents of peptide LK21 in increasing concentrations of TFE. Fifty μM of the peptides were dissolved in H_2_O supplemented with 10%, 20% or 40% TFE and were subjected to circular dichroism measurement. **E.** Circular dichroism spectra and the estimated secondary structure contents of peptide LK21-5W5A in increasing concentrations of TFE, measured as described in C.

Six MPER-containing peptides (Fig 1) ranging from 19 to 39 a.a were prepared with acetylated N-termini and amidated C-termini and were tested as fusion/entry inhibitors against pseudotyped HIV-1(NL4-3). To ensure study the early events of the HIV-1 viral replication cycle a HIV-1 pseudovirus system allowing only a single replication cycle was employed. Pseudovirus particles were produced by the co-transfection of the Env- and Vpu-expression-deficient proviral vector pNLHIVΔuΔss and the Env-, Vpu- and Nef- expressing vector pNL1.5EU+ [48], which generates viral particles capable of entering and infecting target cells but not capable of giving rise to infectious second-generation viral particles. The pseudotyped HIV-1 (NL4-3) particles were pre-incubated with each of the six MPER-containing peptides for 1 h and then added to the target cells, TZM-bl cells, which stably express a tat-responsive luciferase reporter gene allowing for the monitoring of successful HIV-1 entry and viral protein production. The inhibition of infection was monitored by measuring the luciferase expression at 72 h post-infection. Peptide LK21, which contained the entire and exclusively the MPER sequence, inhibited 50% and 80% of viral entry and infection at 8.0 μM and 12.3 μM, respectively (Fig 2A). Inclusion of five HR2 residues to LK21 N-terminus, generating the 26-a.a. peptide QK26, decreased the IC_50_ and IC_80_ values to 3.9 μM and 8.8 μM, respectively. However, further N-terminal extension with the addition of nine and sixteen HR2 residues to LK21, resulting in peptides EK30 and EK37, did not lead to any substantial increase in their anti-viral potency. However, adding the HR2 hydrophilic residues greatly enhanced the solubility and structural stability of the MPER-containing peptides QK26, EK30 and EK37, allowing further application and design, such as peptide dimerization that will be elaborated on in section 2.

To determine the necessity of the conserved MPER C-terminal cholesterol-interacting motif (LWYIK) for the antiviral effect of the peptides, the MPER C-terminal sequence, NWLWYIK, was deleted from the most active peptide, QK26, creating the peptide QT19. This deletion abrogated the antiviral activity, as QT19 failed to inhibit viral infection at concentration up to 33.3μM (Fig 2A). In addition, the importance of the enriched aromatic residue Trp in the MPER region was examined by mutating all the Trp to Ala in the exclusively MPER-containing peptide, LK21, resulting in the peptide LK21-5W5A. Interestingly, the substitutions of all the Trp did not just abolish the antiviral activity of the peptide, but even enhanced viral infectivity in a dose-dependent manner (Fig 2A). At 26.6 μM, the LK21-5W5A increased the viral infectivity by 50%. The inhibitions of viral infectivity by the MPER-derived peptides were not due to cytotoxicity, as incubating TZM-bl cells with 50 μM of the peptides for 24 h did not result in any statistical difference in cell viability between the control (DMSO-treated) and the peptide-treated cells (Fig 2B).

To investigate the structure-function relationship of MPER-containing peptides in inhibiting the entry of pseudo-HIV-1, the secondary structures of the peptides were determined by circular dichroism (Fig 2C). The HR2 region of another class I viral fusion glycoprotein, the spike (S) protein of the severe acute respiratory syndrome associated coronavirus (SARS-CoV), has previously been shown to be largely α-helical and a synthetic peptide (HR2-S) derived from this region served as a control peptide in the following CD study [56]. The peptides were prepared in 10% TFE that mimics the lipidic environment at the juxtamembrane junction. The N-terminal extension of LK21 with HR2-derived residues generally increased the helicity in the MPER-containing peptides. Deleting the C-terminal sequence, NWLWYIK, from QK26 did not change the secondary structure drastically (Fig 2C). To mimic the increasingly lipidic environment transition, which the MPER undergoes during membrane fusion, the TFE concentration was increased from 10%, 20% and to 40%. With increasing TFE concentrations, LK21 gradually exhibited a more alpha-helical conformation, with the first minimum of its spectra shifted from 212 nm to 209 nm, and then to 208 nm, and the estimated α-helical content increased from 41% to 52% and then to 71% (Fig 2D). In contrast, LK21-5W5A, the peptide with all the Trp replaced by Ala, exhibited canonical alpha-helical spectra and a high α-helical content (84%) starting from 20% TFE (Fig 2E), which indicates the importance of Trp residues in maintaining the structural plasticity of the MPER sequence.

### 2. N-terminal dimerization of the MPER-containing peptides selectively enhanced viral inhibition

We next investigated if the antiviral effect of our peptides could be enhanced by dimerization at either the N- or C-terminus. We hypothesized that N-terminally dimerized MPER-containing peptides would mimic the fusion-active oligomerization state of gp41 MPER, thus having an enhanced binding affinity with their interaction partners (e.g. FP), and thereby possess an improved viral inhibitory effect.

**Fig 3:**
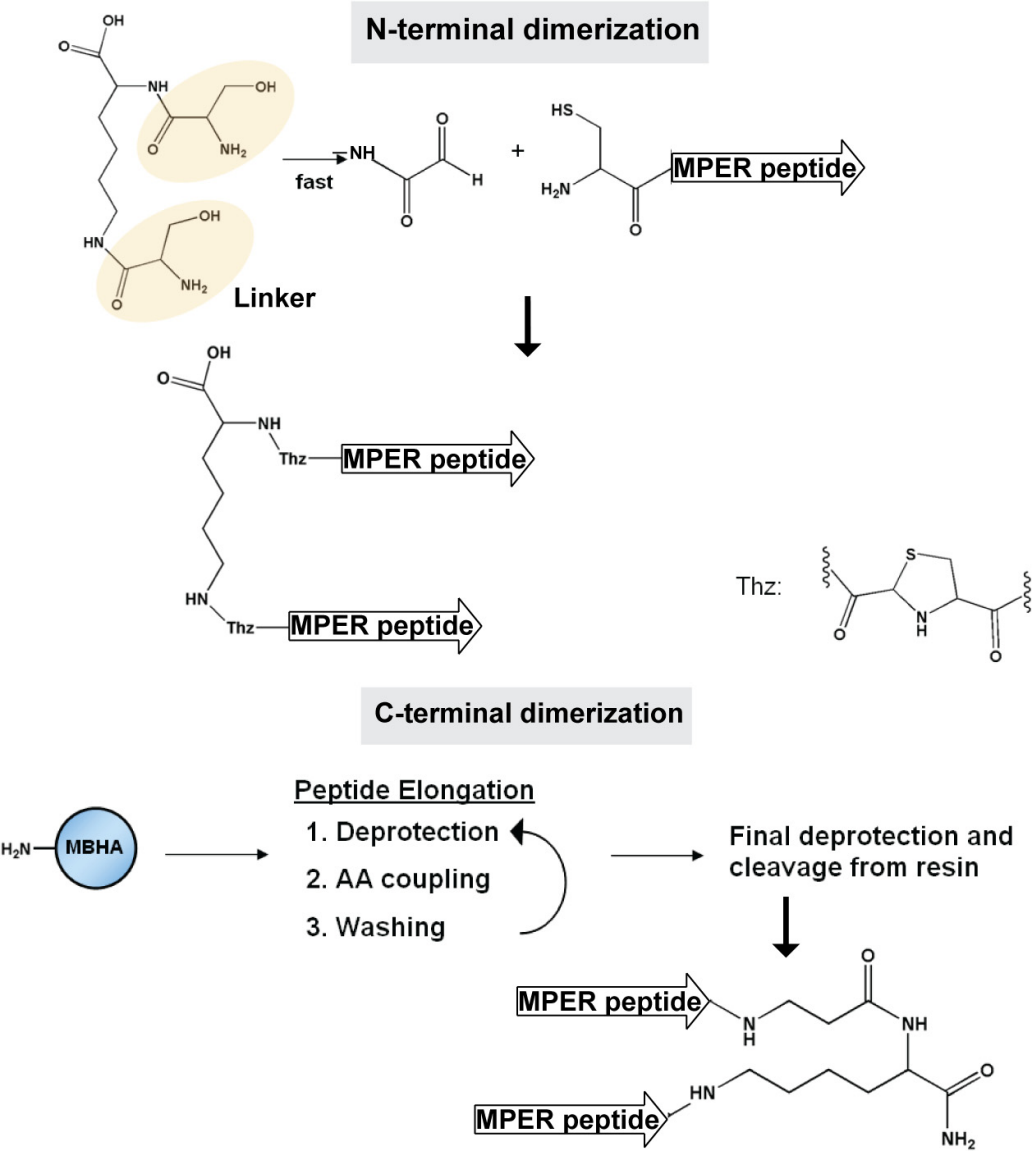
Schematic representation of the peptide N- and C-terminal dimerization strategies. N-terminal dimerization employed a linker molecule consisted of two serine residues branching from a lysine, to which two peptides were attached at their N-termini via a thiazolidine linkage. C-terminal dimerization employed a MBHA resin.

Dimeric peptides were constructed with parallel peptide chains with either two carboxylic termini or two amino termini using chemoselective ligation strategy [57]. To obtain the N-terminal linked dimers (-DN), monomeric peptides were synthesized with an additional N-terminal Cys residue, which was further ligated *via* a thiazolidine linkage to a linker molecule consisting of two Ser branching from Lys (Fig 3). The C-terminal peptide dimers (-DC) were synthesized on MBHA resins, and ligated C-terminally to the linker *via* the amino functional groups on the linker (Fig 3).

**Fig 4:**
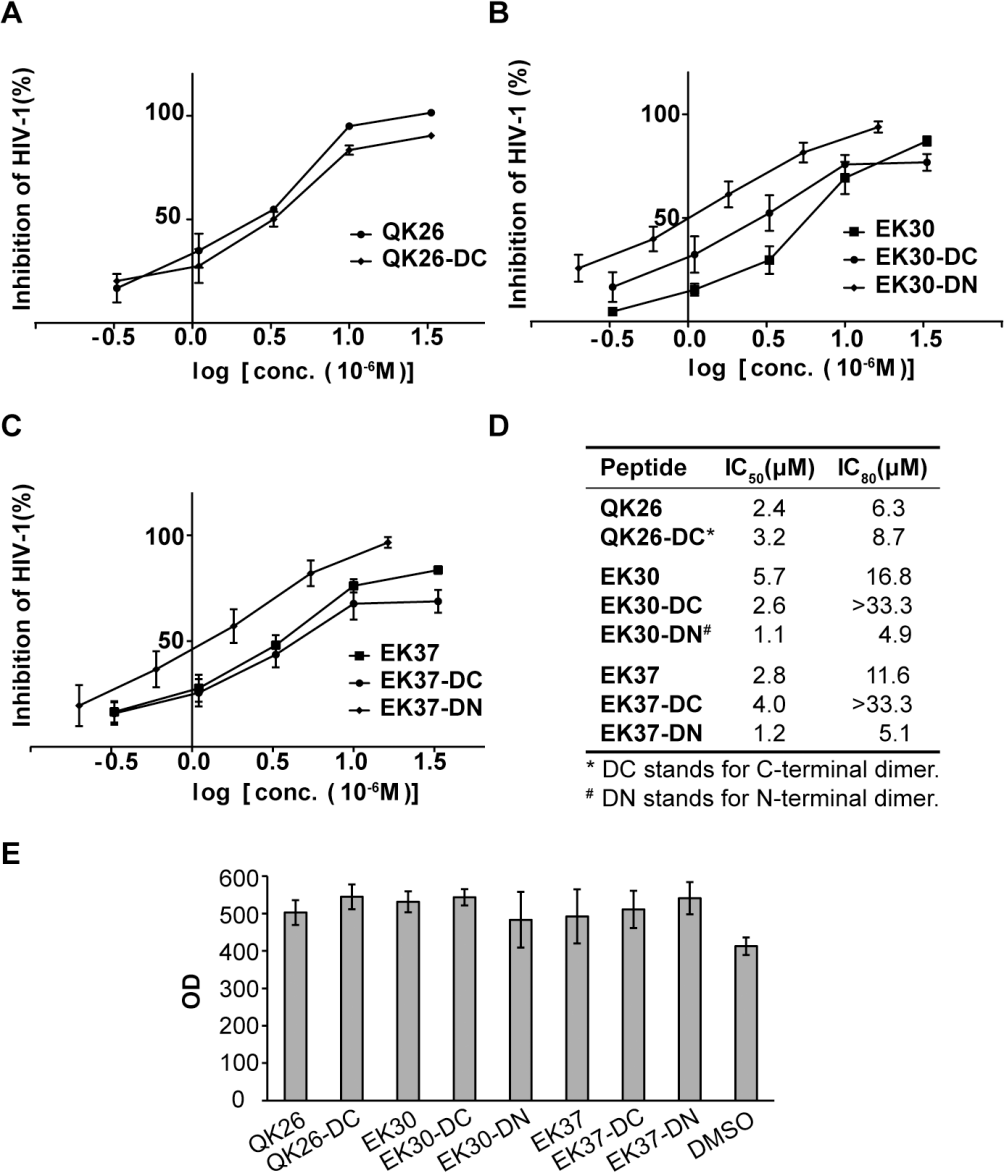
The influence of N- and C-terminal dimerization on the anti-viral effects of the MPER-derived peptides. **A.** Test for inhibition of pseudo HIV-1 (NL4-3) infection of TZM-bl cells by dimerized peptide QK26. **B.** Test for inhibition of pseudo HIV-1 (NL4-3) infection of TZM-bl cells by dimerized peptide EK30. **C.** Test for inhibition of pseudo HIV-1 (NL4-3) infection of TZM-bl cells by dimerized peptide EK37. **D.** Summary of the concentrations of peptides yielding a 50% and 80% reduction in tat-activated luciferase activity, as tested in B, C and D. The concentrations were estimated with GraphPad Prism. **E.** No cytotoxicity effect was observed for the MPER-derived dimeric peptides at 100 μM in TZM-bl cells. Monomeric and dimeric peptides (100 μM) were incubated with 10,000 Vero cells for 24 h. PrestoBlue cell viability reagent was subsequently added to the cells, and cytotoxicity effects were monitored as absorbance values (OD) at 570 nm and 600 nm (baseline).

N- and C-terminal dimers were synthesized from the peptide EK30 and EK37. C-terminally linked QK26 dimer was also synthesized, but due to synthetic difficulties, its N-terminal dimer was not obtained. The dimeric peptides were tested for their ability to inhibit viral entry using the single-round infectivity assay with TZM-bl cells as described above for the monomeric peptides (Fig 4A–4D). The N-terminal dimerization of EK37 and EK30 enhanced their anti-viral potencies (Fig 4B and 4C). The EK37-DN and EK30-DN have IC_50_ values of 1.2 μM and 1.1 μM, an increase of the potency by 5.2- and 1.9-fold compared to the monomeric EK30 and EK37, respectively (Fig 4D). In contrast, the C-terminal dimerization decreased the potency of EK37 and QK26, with IC_50_ values of EK37-DC and QK26-DC elevated by 1.7 and 1.3 fold, respectively (Fig 4D). Meanwhile, the C-terminal dimerization had inconsistent effect on the antiviral potency of the EK30-DC, its IC50 value decreased and IC80 value significantly increased with respect to its monomeric form (Fig 4D). In summary these data indicate that the anti-viral activity is benefited by N-terminal dimerization of MPER-containing peptides. No cytotoxicity effect of the monomeric nor dimeric peptides was observed *in vitro* in TZM-bl cells at concentrations up to 100 μM, as determined by the PrestoBlue cell viability assay (Fig 4E).

### 3. Ala substitutions of MPER Trp residues up-regulate viral Gag protein expression

Our circular dichroism data suggest that the penta-Trp→Ala substitutions induces the MPER peptide to commit to a predominantly helical structure regardless of the environmental lipidity (Fig 2E). This poses the possibility that the same mutations may also affect the secondary structure of the MPER sequence within the HIV-1 precursor Env glycoprotein, gp160, which in turn may disturb its proper folding in the ER, or affect its biosynthesis in other ways and eventually lead to viral defect. To investigate this, site-directed mutagenesis was performed on Env-expressing plasmid pNL1.5EU+ [48] and generated three gp160 mutants, where all five Trp, the three N-terminal Trp (W664, W668 and W670), or the two C-terminal Trp (W676 and W678) in the MPER sequence were substituted with Ala. The resulting constructs were termed W5A, W3A, and W2A, respectively.

**Fig 5:**
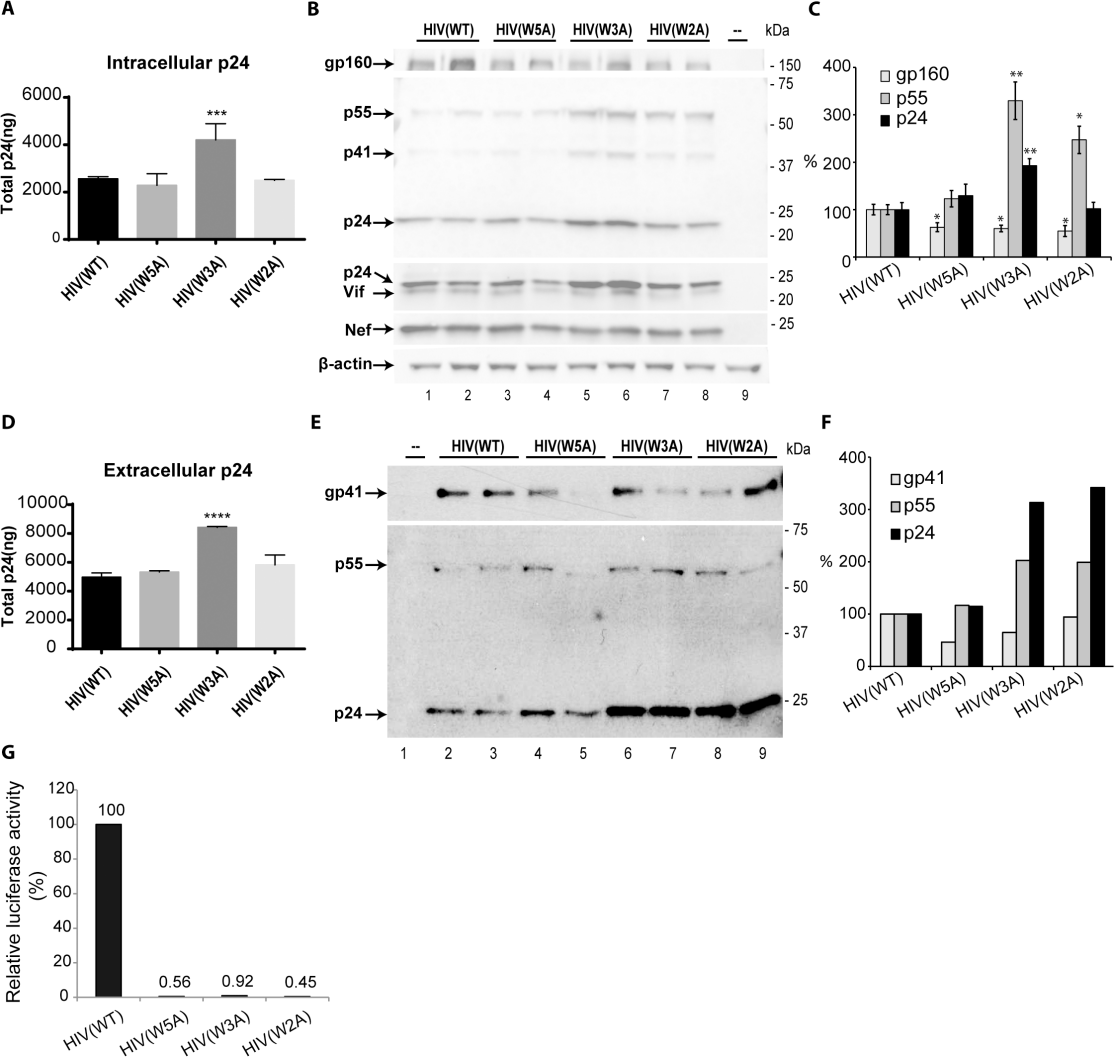
Expression and viral incorporation of viral structural proteins in the context of pseudotyped HIV-1. **A.** p55/Gag-derived p24 levels in pseudovirus-producing HLtat cells. HIV(WT), HIV(W5A), HIV(W3A) and HIV(W2A) were produced by co-transfecting HLtat cells with pNLHIVxΔu∆ss and pNL1.5EU+, pNL1.5EU+W5A, pNL1.5EU+W3A, or pNL1.5EU+W2A, respectively. The p24 levels (ng) in cell lysates were quantified by the automated system Architect (Abbott). ***P < 0.001 as compared to WT by the unpaired Student’s t test. **B.** Steady-state intracellular levels of viral proteins in pseudovirus-producing HLtat cells. HLtat cells from A were harvested 48 h post-transfection and the lysates were resolved by SDS-PAGE and immunoblotted with antibodies against gp41, p24, Vif, Nef and β-actin. Vif and Nef expression served as the transfection control. Un-transfected HLtat cells served as negative control. **C.** Densitometric analysis of protein bands in blots from two independent experiments as described in in B was performed in ImageJ and presented as means ± SD, with gp160, p55/Gag, and p24 levels in HIV(WT) standardized to 100%. *P < 0.05; **P < 0.01 as compared to WT by the unpaired Student’s t test. **D.** p24 levels (ng) in the culture supernatants of pseudovirus-producing HLtat cells. p24 levels in the culture supernatant of HLtat cells in A was quantified by the automated system Architect (Abbott). ****P < 0.0001 as compared to WT by the unpaired Student’s t test. **E.** Env gp41, p55/Gag and p24 levels in precipitated HIV(WT), HIV(W5A), HIV(W3A) and HIV(W2A). Viral particles from the cell culture supernatant from A were precipitated, lysed, separated by SDS-PAGE and immunoblotted with antibodies against gp41 and p24. **F** Densitometric analysis of the blot in E was performed in ImageJ and presented as means, with gp41, p55/Gag and p24 levels in HIV(WT) standardized to 100%. **G.** Entry of the cell-free pseudotyped HIV-1 into TZM-bl cells. Cell culture supernatant from A containing HIV(WT), HIV(W5A), HIV(W3A) or HIV(W2A) was clarified through centrifugation and 0.45μm filtration, and applied to 10^4^ TZM-bl cells. Seventy-two h post-infection, tat-activated luciferase activities in the TZM-bl cells were measured and plotted, with the luciferase activity in HIV(WT)-infected TZM-bl cells standardized to 100%.

To examine the effect of the Trp substitutions on Env expression and viral maturation in the context of virus-producing cells and budding viral particles, HLtat cells were co-transfected with the Env- and Vpu-expression-deficient proviral vector pNLHIVxΔuΔss (described above) and with the WT or mutant pNL1.5EU+ to produce pseudo-typed HIV-1 (NL4-3) viral particles containing WT, W5A, W3A or W2A Env. Accordingly, the expressed pseudo-HIV-1 particles were termed HIV(WT), HIV(W5A), HIV(W3A) or HIV(W2A). At 48 h post-transfection, it was found that the Env levels in cell lysates containing HIV(W5A), HIV(W3A) or HIV(W2A) were lower than that in lysate containing the HIV(WT) (Fig 5B), with the steady-state intracellular gp160 levels in cells producing HIV(W5A), HIV(W3A), HIV(W2A) were approximately 60% of that in HIV(WT) (p = 0.038 for W5A, p = 0.020 for W3A, p = 0.026 for W2A, n = 5) (Fig 5C).

More striking effects of Trp mutations were observed in the other viral structural proteins, specifically p55/Gag and its derivative proteins. The HIV-1 genome encodes three structural proteins, which are the precursor protein Env, p55/Gag, and Gag-Pol. While Env is translated into the ER and is transported to the site of viral assembly at the plasma membrane *via* the secretory pathway, the p55/Gag and Gag-Pol are expressed in the cytosol. The two viral cytosolic precursor structural proteins meet up with Env at the plasma membrane where p55/Gag directly or indirectly interacts with cytosolic tail of Env to recruit it into the assembling viral particles [36]. During or after viral budding, the viral protease within Gag-Pol is activated leading to processing of Gag-Pol and p55/Gag. Among the p55/Gag-derived proteins is the capsid protein, p24, which is commonly used to detect viral particle production [58, 59].

Analysis of the p24 levels in cells producing WT or mutant pseudo-HIV-1 revealed that at 48 h post-transfection p24 in cells producing HIV(W3A) was approximately 45% (p<0.0001, n = 7) higher as compared to p24 in cells producing HIV(WT) (Fig 5A). However, the other two mutants, HIV(W5A) and HIV(W2A), showed no increased levels of intracellular p24 (Fig 5A). The analysis was performed using the automated system ARCHIECT (Abbot) accreditated for detection of p24 in human serum. We also performed in-house validation experiments with HIV-1 infected cells in the presence of the protease inhibitor Indinavir to confirm that the system did not detect the p24 within p55/Gag but only the mature p24 once processed from its precursor protein. The corresponding cell lysates were therefore subjected to analysis by Western blot and the findings clearly confirmed the elevated intracellular levels of p24 in the HIV(W3A) as compared to the HIV(WT) (Fig 5B). Two other viral proteins, Vif and Nef were blotted and served as control for the transfection efficiency to which p55/Gag and p24 expression was standardized. The results further indicated that the intracellular elevation of p24 by 93% (p = 0.01, n = 5) was a result of increased expression of p55/Gag, as the p55/Gag levels were 330% in the HIV(W3A) mutant as compared to that in the HIV(WT) (p = 0.007, n = 5) (Fig 5B and 5C).

The extracellular p24 levels in the HIV(W3A)-expressing cultures, analyzed by the automated system ARCHIECT (Abbot), were further found to be 67% higher than those of the HIV(WT) (p<0.0001, n = 7), consistent with the increased intracellular p24 expression levels from this mutant (Fig 5D). A smaller increase by 10% of extracellular p24 levels were detected in the HIV(W5A) (p = 0.0378, n = 7) expressing cultures (Fig 5D). In addition, the viral particles were isolated from equal volumes of the respective cell culture supernatants and subjected to analysis for Env, p55/Gag and p24 by Western blot followed by densitometric analysis. As compared to that in HIV(WT), there was moderate decrease in Env level among the mutant viral particles. In contrast, p55/Gag and p24 contents in isolated HIV(W3A) virus particle were elevated by approximately 200% and 310%, respectively (Fig 5E and 5F), which is in line with elevated p24 levels detected in both the cell culture lysates and supernatants by the automated system, ARCHITECT. It further indicates an increased HIV(W3A) viral particle production as a result of increased intracellular p55/Gag expression. Similar increase of p55/Gag and p24 contents was observed in the HIV(W2A) particle isolate (Fig 5E and 5F).

The influence of the Trp substitutions to Ala in the MPER on viral entry and infectivity was also tested by adding equal volumes of the respective cell free culture supernatant to the TZM-bl cells. Forty-eight hours post-infection, the Tat-activated expression of luciferase in the TZM-bl cells were measured and showed that in contrast to the HIV(WT), all the mutant particles HIV(W5A), HIV(W3A) and HIV(W2A) lost their ability to infect the TZM-bl cells (Fig 5G).

These data collectively suggest that, while substitutions of the Trp to Ala in the MPER sequence of gp160 decreased its intracellular expression levels and consequently moderately reduced the Env incorporation into the viral particles, the mutations significantly influenced the intracellular expression levels of p55/Gag. In particular, the substitutions of the Trp664, Trp668 and Trp670 in the MPER significantly elevated the intracellular p55/Gag expression and subsequently the viral particle production. Furthermore, the substitutions of the Trp to Ala rendered the viral particles non-infectious, in accordance with the previous literature [17].

## Discussion

The gp41 MPER (HIV-1_NL4-3_: LDKWASLWNWFNITNWLWYIK) induces membrane-fusion-required membrane perturbation in the viral envelope and cellular membranes, through its two conserved membrane-active sequence elements; the enrichment of aromatic residues (e.g. Trp) and a C-terminal cholesterol interacting motif (LWYIK) [17]. Here we show that the membrane active sequential elements of gp41 MPER are vital for MPER-containing short peptidyl fusion inhibitors, as the omission of the C-terminal motif (LWYIK) and the penta-Trp→Ala substitution abrogated their anti-HIV-1 activity. The peptide anti-viral activity could be enhanced through N-dimerization, but not C-dimerization.

In this study, peptide LK21, containing the entire MPER sequence, inhibited pseudotyped HIV-1(NL4-3) entry and infection with the IC_50_ values at 8.0 μM. N-terminal extension of LK21 with five amphipathic residues derived from HR1-biding region of HR2 (628 a.a.–666 a.a.) enhanced the anti-viral effect and reduced the IC_50_ by half. Further N-terminal extension of LK21 did not enhance its anti-viral effect substantially, probably because this sequence does not include enough residues to mediate a stable interaction between the peptide and the viral HR1 as 6-HB formation. Instead, the enhancement of the anti-viral efficacy through addition of five amphipathic residues correlated with the subtle increase in the helicity of the resulting peptides while maintaining the general structural profiles, as estimated from the respective circular dichroism spectrum. In the context of gp41, this amphipathic sequence upstream to the MPER serves as an extension into HR2 and induces the N-terminus of MPER in fusogenic gp41 to transform from an extended conformation to a helix upon increases in local lipidity [60]. This suggests that the addition of five residues of the N-terminal amphipathic sequence confers the peptides a more stable conformation and a capacity to interact with HR1, which lead to their enhanced anti-viral efficacy.

The formation of 6-HB has been suggested to induce not only a secondary structural transformation in the MPER N-terminus, but also to result in its quaternary structural rearrangement and oligomerization [60, 61]. Meanwhile, the MPER C-termini of the neighboring gp41 stay monomeric and assume an extended platform to destabilize the cholesterol-enriched lipid bilayers, likely through its CRAC motif (LWYIK) [24–26]. Our data show that deletion of the C-terminal sequence including the conserved CRAC motif in the MPER-containing peptide QK26 abrogated its anti-viral effect, suggesting that this membrane-active sequence plays an essential role in the anti-viral mechanism of the peptide. The peptide dimerization data further support that the inclusion of free C-termini for membrane interaction in the MPER peptides are important for their inhibition of HIV-1 entry, as constraining the peptide C-termini through C-terminal resulted in the anti-viral effects of peptide EK37 and QK26 unenhanced. A previous study by Nomura *et*.*al* also observed that the C-terminal trimerization of T20 failed to enhance its anti-viral efficacy significantly, presumably because the trimerization constrains its membrane-active MPER sequence, offsetting the activity enhancement due to the potential cooperative interaction between its HR2 sequences and the viral gp41 HR1 [38, 62]. The enrichment of Trp residues in MPER is a second important membrane-active characteristic exhibited by class I fusion glycoproteins [63]. Trp contains a large indole-ring side-chain that is preferred by the juxtamembrane interface of proteins, facilitating protein-membrane interaction and stabilizing protein structure [64–66]. In this study, we examined the influence of Trp residues in the design of fusion inhibitors by substituting the indole-ring side-chains of the five Trp residues in LK21 with the alkyl moieties of Ala. Surprisingly, the resulted LK21-W5A peptide promoted viral infectivity rather than inhibiting it, a phenotype that has been also observed when Ala-substituting the Trp residues of the SARS-CoV MPER peptide [67]. The data indicate the significance of membrane-active elements in MPER-containing peptides, both the CRAC motif and Trp residues, for the inhibition of HIV-1 entry. This is in agreement with previous findings that increased membrane reactivity owing to inclusion of the MPER N-terminal sequence (LDKWASLWNWF) as in T20 and T1249, correlated with an enhanced anti-viral effect of the peptides [29–32]. Of note, our data further demonstrate the involvement of the C-terminal CRAC motif (LWYIK) in facilitating viral inhibition for short MPER-containing peptides with minimal inclusion of HR2 sequences.

Aside from its involvement in membrane fusion events, Trp residues have also been shown to modulate the interaction between MPER and other viral domain(s) during viral entry. Specifically, gp41 MPER interacts with FP to form a continuous hydrophobic track along with the 6-HB and promote membrane juxta-positioning [68, 69]. Shortly before the interaction stabilizes and during the transition from the pre-fusion to post-fusion conformation, the MPER and/or FP could be temporarily exposed and vulnerable to dominant-negative binding by peptidyl fusion inhibitors, such as LK21 of this study. We have recently shown that MPER in the SARS-CoV spike protein interact with the internal FP (IFP) in a Trp-dependent manner [67]. In the same study, Trp→Ala substitutions also resulted in MPER-containing peptides to lose the dose-dependent inhibition of coronavirus entry, correlating with the disruption of the MPER-IFP interaction. In gp41, Trp670 has previously been shown to mediate the MPER-FP interaction [70]. Its Ala substitution in the peptide LK21-5W5A could lead to a diminished affinity between the peptide and the FP in gp41, and hence contribute to the loss of the anti-viral effect of the peptide. Furthermore, penta-Trp→Ala substitutions in LK21 prematurely predisposes the peptide to the final helical conformation, losing the lipidity-induced structural plasticity. It suggests that the potential interactions between MPER and other viral regions (such as FP) could also be conformation-dependent, and that the transition of MPER from an extended to helical conformation could result in an intermediate species for such interaction to take place.

Whilst the C-terminal dimerization failed to enhance the anti-viral efficacy of peptides studied here, N-terminal dimerization lowered their IC_50_ and IC_80_ values up to 5-fold. Our data would suggest that N-terminally dimerized MPER-containing peptides mimic the fusion-active oligomerization state of gp41 MPER, thus enhancing binding affinity with their interaction partners (e.g. FP and membrane), thereby having an improved anti-viral effect. The optimal length of the linker between the monomers could be explored to enhance the flexibility of the unit peptide, which may further enhance the cooperative interactions between the peptide multimer and gp41. The differential effects of N- and C-dimerization on the anti-viral effects of MPER-containing peptides have been previously observed in our group, with SARS-CoV as the model virus (S3 Fig). N- and C-terminally dimerized peptides containing the S protein MPER sequence were prepared as described in this paper, and enhanced and inhibited, respectively, the anti-viral effects against pseudotyped SARS-CoV compared to the monomeric peptide.

The HIV-1 MPER contains neighboring epitopes for broadly neutralizing antibodies, including 662-DKWA-665 for antibody 2F5 and 669-NWFNIT-674 for antibody 4E10 [71, 72]. Both antibodies have been shown to neutralize different strains of primary isolates of HIV-1 when administrated in cocktails in animal models [73]. Despite being proven difficult, a tetramer peptide mimetic containing the MPER sequence has elicited broadly neutralizing antibodies 4E10 and 2F5 in guinea pigs [74]. This rises the potential immunogenicity problem with the MPER-derived anti-viral peptides. However, any such immune responses is not expected to induce major adverse effects in host, as any elicited anti-HIV-MPER antibodies could probably be immunologically tolerated by the host, and may even further help to control the HIV replication. The direct evidence of the host immunological tolerance of 2F5 and 4E10 has been provided their recognition of two autoantigens, human kynureninase and splicing factor 3b subunit 3 [75]. Furthermore, T20 which contains the epitopes for 2F5 and 4E10 has not only been clinically proven to be safe and effective in the presence of cross-reactive antibodies [76], it has been shown to act synergistically with 4E10 in inhibiting viral infectivity [72]. In addition, the neutralizing capacities of the antibodies 2F5 and 4E10 require functional Env trimers and probably would not neutralize the MPER-derived peptides’ anti-viral effect [73]. Nevertheless, at the prospect of developing MPER-derived peptidyl entry inhibitors, any potential elicited immunological responses should be examined and investigated [77]. Finally, the peptides should be tested with different subtypes of HIV-1 to confirm the antiviral activity.

Env interacts with host membranes during both fusion/entry, and its biosynthesis and trafficking during viral budding. The substitution of all five Trp to Ala in the MPER antiviral peptide resulted in it predominantly adopting a helical structure regardless of the environmental lipidity. This suggest that the Trp residues could be equally vital for the secondary structure of the MPER region within gp160, and consequently their substitution could hamper a proper folding and function of gp160. We further investigated the biological relevance of the MPER Trp residues in the biosynthesis of Env and other viral proteins. Previously, Salzwedel *et al*. found that Trp mutations in the MPER affected incorporation of Env into virions but no effect of the mutations was seen on the Env levels in the cell lysate or on the plasma membranes [17]. Here we observed that, in the transfected HLtat cells expressing pseudotyped HIV-1, Ala substitutions of all five Trp, the N-terminal three Trp (W664, W668, W670), and the C-terminal two Trp (W676 and W678) residues, although moderately, lowered the steady-state intracellular gp160 and intraviral gp41 levels without affecting the migration patterns as compared to WT. The discrepancy between their and our findings may be due to differences in the assay used to quantify Env, immunoprecipitation using patient serum versus immunoblotting.

More interestingly, we observed that the mutations in Env up-regulated the expression of the capsid protein p24 through up-regulating its precursor protein p55/Gag, despite differential biosynthesis pathways between p55/Gag and Env. The results generally agreed with the previous understandings that Env expression inhibits the steady-state intracellular level of p55/Gag. It has been shown that the downregulation of p55/Gag expression by Env could be executed at both protein level through the Env cytoplasmic tail, or at RNA level through actions *via* the Rev response element within the *env* gene [78, 79]. In this study, the upregulation of Gag/p55 protein intracellular and intraviral levels were not proportional to the reduction of Env protein levels and interestingly the pseudo-HIV-1 in which the N-terminal three Trp or the C-terminal two Trp were substituted gave a much higher increase of p55/Gag than the pseudo-HIV-1 with all five Trp replaced with an Ala. Hence it remains an open question if there is another distinctive mechanism through which the Trp→Ala mutations in Env MPER upregulated the Gag expression, besides through lowering the intracellular Env levels.

While Env is responsible for receptor/co-receptor recognition, membrane fusion and viral entry; p55/Gag can independently induce the assembly and budding of virus-like particles in living cells and *in vitro*. Its derivative protein, p24, dictates the proper maturation, size and morphology of the budding virions, which are essential for viral infectivity [80]. Our data indicate that the Trp residues in Env MPER are important for the biosynthesis of Env and another major viral structural proteins p55/Gag, which could collectively affect the viral fitness and be an additional factor, besides the absence of membrane-active Trp indole ring sidechain, for the failed viral entry, as observed in a previous study [17] and confirmed in this study.

## Conclusions

In summary, our findings suggest active participation of membrane-active elements within MPER (e.g. Trp) in events that require protein-membrane interactions during both viral entry and assembly. These results indicate the importance of the five Trp residues and C-terminal sequence (NWLWYIK) in MPER for the design of future MPER-based fusion inhibitors and offer further insights into the understanding of viral structural proteins biosynthesis. The role of gp41 MPER Trp residues in modulating the viral contents of Gag proteins might guide the discovery of potential therapeutic targets against HIV-1 infection.

## Supporting Information

S1 Fig Trafficking of the SARS-CoV spike protein to the lipid rafts. 293T cells were transiently transfected to express wild type spike protein (Swt). Twenty-four h post-transfection, cells were harvested and lysed on ice in 1% Triton X-100 TNE lysis buffer, and the cell postnuclear extracts were fractionated by 5%-30% sucrose gradient ultracentrifugation. Eleven fractions were collected from top to bottom after centrifugation. Samples were resolved by SDS-PAGE and western blot, with or without PNGase F treatment. Caveolin-1 serves as a positive marker for lipid raft.
(TIF)

S2 Fig Effect of Trp→Ala mutation on the trafficking of SARS-CoV spike protein to the lipid rafts S3w3a. The triple Trp→Ala substituted mutant of Swt was expressed in 293T cells and the lipid raft of the transfected cells were extracted, as described in S1 Fig Both Swt and S3w3a were detected in the lipid-raft-containing interfacial section between 5% sucrose and 30% sucrose, co-localizing with the lipid raft marker caveolin-1. Both constructs contain two protein species with different sizes of 180 kDa (mature) and 170 kDa (immature), due to different glycosylation and maturation stages [81]. For both Swt and S3w3a, N-deglycosylation *via* PNGase F confirmed the gp180 and gp170 species originated from a common precursor but differed in glycosylation stage. The majority of Swt gp180 was directed to lipid-raft containing fractions, while Swt gp170 was predominantly retained in the bottom fractions. Triple Trp→Ala substitutions resulted in an altered trafficking pattern of the mature form of the S protein. In S3w3a, both S3w3a gp180 and S3w3a gp170 were found in the upper and bottom fractions at equal amounts, suggesting that a lower percentage of mature S3w3a was recruited to the lipid raft. The data suggest that the Trp residues function to fine-tune the clustering of fully mature S protein into lipid rafts during budding.
(TIF)

S3 Fig Effects of N- and C-dimerization on the anti-viral effects of peptides containing SARS-CoV spike MPER. Peptide M_SARS,_ a peptide containing the SARS-CoV S protein MPER sequence (KYEQYIKWPWYVWLGF) and its N- and C-terminal dimers, N-M_SARS_ and C-M_SARS_, were tested as fusion inhibitors against pseudotyped SARS-CoV. Pseudotyped SARS-CoV was prepared by co-transfecting 293T cells using calcium phosphate transfection method with pNL4-3Luc+Env-Vpr- and pcDNA3.1-OPT9-S mutant plasmids. pNL4-3Luc+Env-Vpr- was kindly provided by Prof. Zhang Linqi (Aaron Diamond AIDS Research Center, Rockefeller University, New York 10016). Peptides were incubated with the virus for 1 h under 5% CO_2_ at 37°C, prior to being added to Vero E6 cells and incubated for another 72 h. Inhibitory activities of the peptides were calculated from the luciferase activities of the Vero E6 cells, determined by a TD-20/20 Luminometer (Tuner Designs).
(TIF)
