# Supplementary figures and images for: Membrane-Active Sequences within gp41 Membrane Proximal External Region (MPER) Modulate MPER-Containing Peptidyl Fusion Inhibitor Activity and the Biosynthesis of HIV-1 Structural Proteins
Source: PLoS One. 2015 Jul 31;10(7):e0134851. doi: 10.1371/journal.pone.0134851 (PMC4521866; doi:10.1371/journal.pone.0134851)

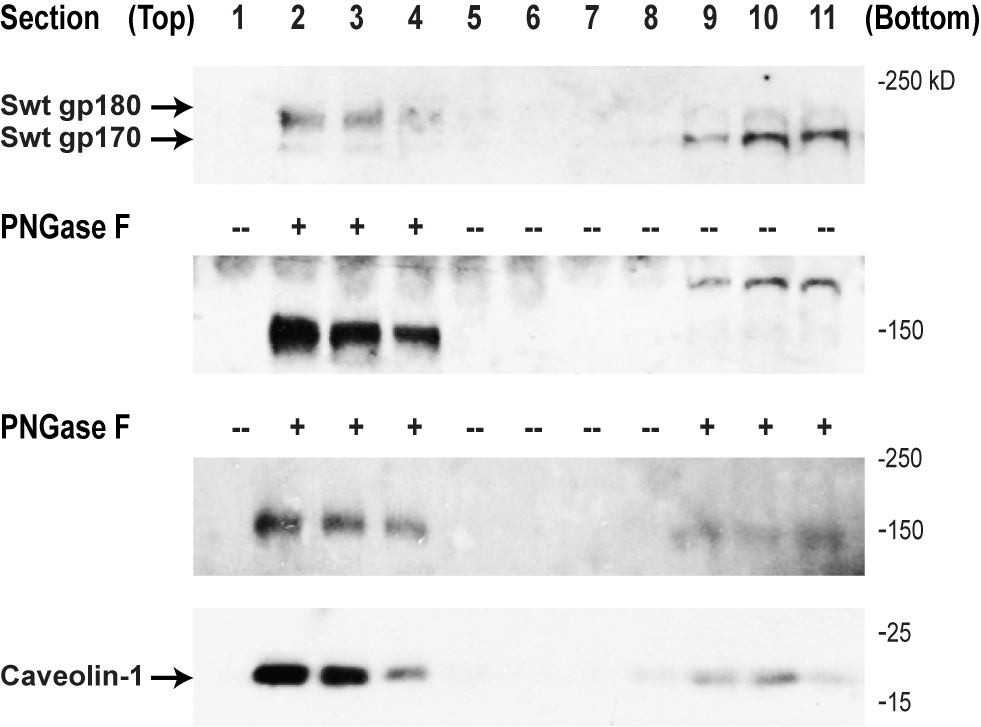

Supplement: S1 Fig — 293T cells were transiently transfected to express wild type spike protein (Swt). Twenty-four h post-transfection, cells were harvested and lysed on ice in 1% Triton X-100 TNE lysis buffer, and the cell postnuclear extracts were fractionated by 5%-30% sucrose gradient ultracentrifugation. Eleven fractions were collected from top to bottom after centrifugation. Samples were resolved by SDS-PAGE and western blot, with or without PNGase F treatment. Caveolin-1 serves as a positive marker for lipid raft. (TIF) [file pone.0134851.s001.tif]

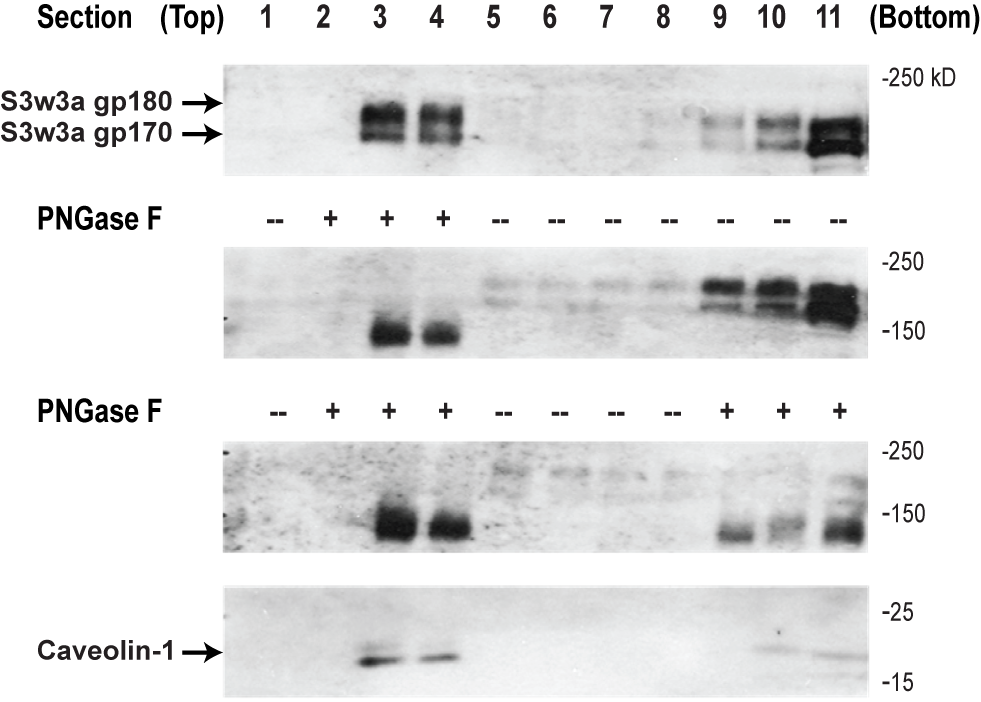

Supplement: S2 Fig — The triple Trp→Ala substituted mutant of Swt was expressed in 293T cells and the lipid raft of the transfected cells were extracted, as described in S1 Fig Both Swt and S3w3a were detected in the lipid-raft-containing interfacial section between 5% sucrose and 30% sucrose, co-localizing with the lipid raft marker caveolin-1. Both constructs contain two protein species with different sizes of 180 kDa (mature) and 170 kDa (immature), due to different glycosylation and maturation stages [81]. For both Swt and S3w3a, N-deglycosylation via PNGase F confirmed the gp180 and gp170 species originated from a common precursor but differed in glycosylation stage. The majority of Swt gp180 was directed to lipid-raft containing fractions, while Swt gp170 was predominantly retained in the bottom fractions. Triple Trp→Ala substitutions resulted in an altered trafficking pattern of the mature form of the S protein. In S3w3a, both S3w3a gp180 and S3w3a gp170 were found in the upper and bottom fractions at equal amounts, suggesting that a lower percentage of mature S3w3a was recruited to the lipid raft. The data suggest that the Trp residues function to fine-tune the clustering of fully mature S protein into lipid rafts during budding. (TIF) [file pone.0134851.s002.tif]

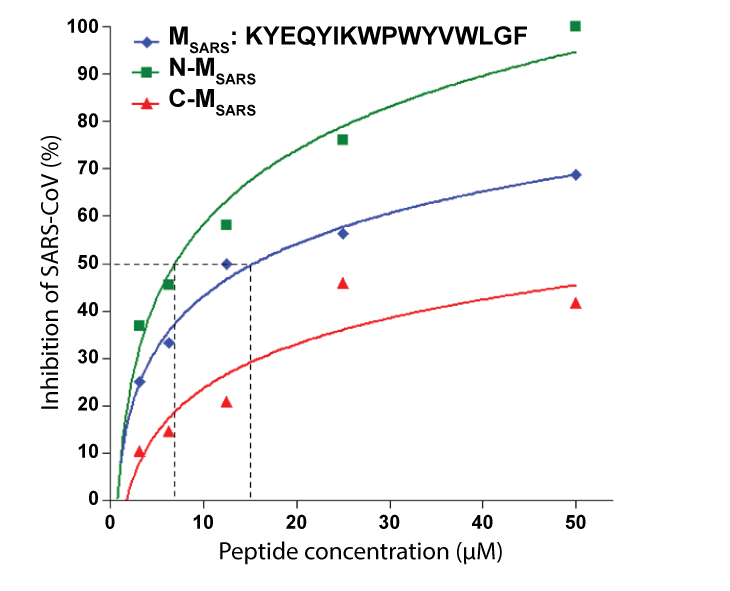

Supplement: S3 Fig — Peptide MSARS, a peptide containing the SARS-CoV S protein MPER sequence (KYEQYIKWPWYVWLGF) and its N- and C-terminal dimers, N-MSARS and C-MSARS, were tested as fusion inhibitors against pseudotyped SARS-CoV. Pseudotyped SARS-CoV was prepared by co-transfecting 293T cells using calcium phosphate transfection method with pNL4-3Luc+Env-Vpr- and pcDNA3.1-OPT9-S mutant plasmids. pNL4-3Luc+Env-Vpr- was kindly provided by Prof. Zhang Linqi (Aaron Diamond AIDS Research Center, Rockefeller University, New York 10016). Peptides were incubated with the virus for 1 h under 5% CO2 at 37°C, prior to being added to Vero E6 cells and incubated for another 72 h. Inhibitory activities of the peptides were calculated from the luciferase activities of the Vero E6 cells, determined by a TD-20/20 Luminometer (Tuner Designs). (TIF) [file pone.0134851.s003.tif]
